# Correction: Evaluation of an Intervention to Improve Skills in Diagnostic Radiology of Rural Physicians over One Year in Four Rural Hospitals

**DOI:** 10.1371/journal.pone.0103166

**Published:** 2014-07-15

**Authors:** The PLOS ONE Staff

## Notice of Republication

This article was republished on June 27, 2014, due to the exclusion of Figure 1. Please download this article again to view the correct version. The originally published, uncorrected article and the republished, corrected article are provided here for reference.

## Supporting Information

File S1 Originally published, uncorrected article.
(PDF)

File S2 Republished corrected article.
(PDF)
