# Supplementary material for: Correction: Evaluation of an Intervention to Improve Skills in Diagnostic Radiology of Rural Physicians over One Year in Four Rural Hospitals
Source: PLoS One. 2014 Jul 15;9(7):e103166. doi: 10.1371/journal.pone.0103166 (PMC4099371; doi:10.1371/journal.pone.0103166)
Supplement: File S2 — Republished corrected article. (PDF) [file pone.0103166.s002.pdf]

Evaluation of an Intervention to Improve Skills in Diagnostic Radiology of Rural Physicians over One Year in Four Rural Hospitals

Tienan Feng[1,3]☯, Xiwen Sun[2]☯, Wenxin Niu[3], Hengjing Wu[3], Chenghua Jiang[3]*

1 School of Life Sciences and Technology, Tongji University, Yangpu, Shanghai, China, **2** Shanghai Pulmonary Hospital affiliated to Tongji University, Yangpu, Shanghai, China, **3** Department of Disaster and Emergency Medicine, Eastern Hospital, Tongji University School of Medicine, Pudong, Shanghai, China

Abstract

Background: Primary health care and patient triage are two basic functions of rural hospitals. As a routine test, the diagnostic radiology is still unavailable in some rural hospitals in China. Therefore, high-level hospitals are often the first choice of rural residents when they feel unwell. It brings serious social problems. This study was designed to propose an on-the-job drilling schema with integration of practical medical recordings and experienced radiological doctors as tutors to improve skills in diagnostic radiology of rural physicians.

Methods: The information technology was used to help the contact between rural doctors and tutors. In a longitudinal pre/post-test control study design, a cohort of 20 young physicians, each of whom was working in a rural hospital and had a work experience less than two years, were established as the trial group over one year. Another 20 similar counterparts were established as the control group. Participants' performances were evaluated in four categories at five-time point (TP).

Results: The trial group significantly outscored the control group on the style of writing at the second TP (d = 2.28); on the accuracy of the image description at final TP (d = 1.11); on the accuracy of the diagnosis at the fourth TP (d = 3.62); and on the correct treatment selection at the third TP (d = 6.45). The aspects with the most improvement were the accuracies of the diagnosis and the treatment selection.

Conclusion: This study provided the detailed evidences that applying the on-the-job drilling schema has a significant effect on the skills improvement in diagnostic radiology of rural physicians. It was also concluded that the educational intervention based on practical cases was better than that only based on didactic slides presentation.

Citation: Feng T, Sun X, Niu W, Wu H, Jiang C (2014) Evaluation of an Intervention to Improve Skills in Diagnostic Radiology of Rural Physicians over One Year in Four Rural Hospitals. PLoS ONE 9(4): e93889. doi:10.1371/journal.pone.0093889

Editor: Zhaohua Ding, Vanderbilt University, United States of America

Received July 31, 2013; **Accepted** March 11, 2014; **Published** April 4, 2014

Copyright: © 2014 Feng et al. This is an open-access article distributed under the terms of the Creative Commons Attribution License, which permits unrestricted use, distribution, and reproduction in any medium, provided the original author and source are credited.

Funding: This work was supported by integrated demonstration of rural remote areas disaster early warning and key rescue technology (NC2010RD0080), and The National Key Technology R&D Program (Grant No. 2014BAL05B00). The funders had no role in study design, data collection and analysis, decision to publish, or preparation of the manuscript.

Competing Interests: The authors have declared that no competing interests exist.

* E-mail: jch@tongji.edu.cn

☯ These authors contributed equally to this work.

Introduction

Public medical service is an essential part of either an urban or a rural region. The rapid economic growth of China lifted half a billion people out of poverty [1], but significant economic disparities have also emerged [2]. Massive developmental gaps exist between urban and rural regions [3,4]. The vast investment in urban regions attracted most talented physicians into urban top-level hospitals. As a result, the outstanding medical resources attract patients across the nation. The patient number and guidance from experienced tutors are two important factors for improvement of the physicians' clinical ability [5]. Rural doctors might meet various unintentional obstacles to these two factors. Several continuing medical education (CME) projects implemented to address the issue and many rural physicians indeed were trained, but the benefits were not obvious. Still, there are few methods that can help rural physicians to improve their diagnostic skill efficiently and effectively.

Most rural physicians who did not graduate from reputed medical universities or colleges do not develop solid knowledge of medicine. They often could not diagnose many illnesses independently. It is very hard for them to become independent in their current work environments. Though there are some internet resources that can help them acquire quite a bit of knowledge of medicine [6,7], rural physicians prefer on-site clinical skills training [8,9]. One efficient way for them is to learn in a top-level hospital. However, off-the-job training for some time is nearly impossible due to shortage of staff and insufficient funds.

Practical skills are what rural doctors need most. A more useful way is to drill those skills. Unlike training, the term 'drilling' refers to the process where some skill is repeated many times to help an individual master it. In the consideration of rural physicians' educational background and work condition summarized from on-

site survey, the study proposed a simple on-the-job drilling schema based on the integration of practical medical recordings which were used to construct digital stimulated patients (DSP) and experienced radiological doctors as tutors to improve skills in diagnostic radiology of rural physicians. In our view, the schema belongs to the area of Personal Development Training and only covers the part of skill training. In addition, it seems that our method can also be a practical part of Foundation Training, Maintenance or Refresher Training, On-the-Job Training and Induction Training. With the advance of information and communications technology (ICT), such as the Internet, the personal computer, and the smart phone [10], many medical informations can be easily acquired. Researchers have reported on methods of using E-learning and distance education through the support of ICT devices [7,11–14]. In our study, rural doctors could get the DSPs and frequently discuss questions with tutors via the information technique. We thought this schema could be effective to improve the skill level of rural physicians. Diagnostic radiology is an essential test, but was not satisfactorily mastered by most rural physicians. Therefore, this study, based on the on-the-job drilling schema, was designed to make an exploratory research on improving skills in diagnostic radiology of rural physicians.

Compared to current education [15], the DSP intervention based on practical medical recordings [16] and the drilling process was designed to stimulate the real atmosphere in radiology rooms in top-level hospitals. The outcomes of this project were evaluated by learning outcomes and performance improvement [17] ascertained from the written reports from participants. These reports were graded in four areas: the appropriate formatting of the report, the accuracy of the description of the radiographic images, the accuracy of the diagnosis, and the appropriate selection of subsequent treatment for the DSP. This study established five time points (TPs) for recording the performance of participants. A generalized mixed-efforts model was used to analyze the change in the physicians' performances and to assess the impact on each part.

The drilling mode proposed in this study is a form of simulation-based medical education (SBME) [18] and could be used as a part of CME for the rural physicians. SBME has been used for several decades. It is necessary to modify SBME when it is used in different subareas [19]. This study was aimed to explore the use of this modified SBME method and evaluate its effectiveness and efficiency in the improvement of rural physicians' education [20].

Methods

Ethics statement

All data used in this project was anonymous. Before this study, all participants were provided with a statement on the start-up screen of the system, which specified the research procedure. Participants gave their informed consent both verbally and by clicking a response button that asked for their approval to participate in the trial. As anonymity was vital for the success of the study, this procedure ensured that no record would link their identity to this research. The ethics committees of medicine and life sciences, Tongji University approved this consent procedure (No. 2010-131).

Participants

The participants were selected randomly from four rural hospitals. All of them had less than two years of work experience. A cohort of 20 participants who agreed to participate was enrolled in the trial. Another cohort of 20 rural physicians was enrolled as the control group. All participants' original performance was evaluated before the study. Five TPs were selected to record the performance results of both cohorts. Performance evaluated at the first TP is the original state of participants. For the training group, the recordings were to evaluate the efficiency of the drilling schema. The effectiveness of the drilling schema was compared between two groups.

Digital stimulated patients and tutors

To offer the participants the two positive factors mentioned above, we created the DSP and designed the asynchronous tutor assistance schema. DSPs were built from real medical recordings in top-level hospitals and the recordings were reviewed by experts. In this project, most DSPs with obvious symptom were designed as positive ones, while some healthy people were designed as negative DSPs. 1,100 DSPs were built and divided into 5 groups (A, B, C, D and E). 250 DSPs were distributed from group A to D. Except the first TP, participants in drilling group received one group of DSPs before each TP test, in the order from A to D. Group E contained 100 DSPs. At each TP, 20 DSPs were sent from Group E to evaluate the diagnostic performance of both groups. When the participants encountered problems during the drilling process, they could submit questions to the tutors via Internet. Participants were not encouraged to contact with tutors at the beginning. After they diagnosed DSPs, they had read the standard reported written by these tutors or their colleagues. Though reading these reported, participants could know the general annotation of lexicons, which help them master key points to communicate with tutors.

Drilling scenario

Physicians in rural hospitals did not serve many patients on one weekday and most of the patients preferred to see their physicians in the morning. Therefore, a significant amount of time was indeed available while the physicians were on duty. The scenario designed in this study was not only to help rural physicians improve their diagnostic skills but also to standardize their diagnostic habit. The scenario was a part of the general clinical process in top-level hospital (Figure 1). This project was divided into 5 periods. In the first period, all participants were evaluated before the drilling training began. Then, participants in training group completed the drilling process by themselves at each period. After they diagnosed a DSP, the rural physicians could see the diagnosis results completed by a radiologist from top-level hospitals. Besides the accuracy of the diagnosis, we hoped that learning a standardized writing style and careful descriptions would also be beneficial to help avoid medical mistakes. An e-investigator also recorded the amount of time that each participant spent on drilling. If the time that a physician spent on completing the drilling exercises was less than other participants did greatly, he/she was either placed in the control group or eliminated from the trial. At each end of one period, there was a test. When the test finished, the participants in trail group and the tutors discussed any questions and difficulties regarding diagnostic radiology. The drilling schema was built on a beta platform mainly based on Microsoft Server 2005 and Microsoft Silverlight.

Performance Evaluation

The performance of participants was evaluated in the format of the written report, the accuracy of the image description, the accuracy of the diagnosis, and the correct treatment selection. All test reports were scored anonymously by experts. The score of a term for perfection is 1 point. If the incongruence reflected on the score, the average method was used. The scores were compared between the two groups using t-test or rank sum test. Furthermore, analysis of covariance (ANCOVA) was conducted to compare the

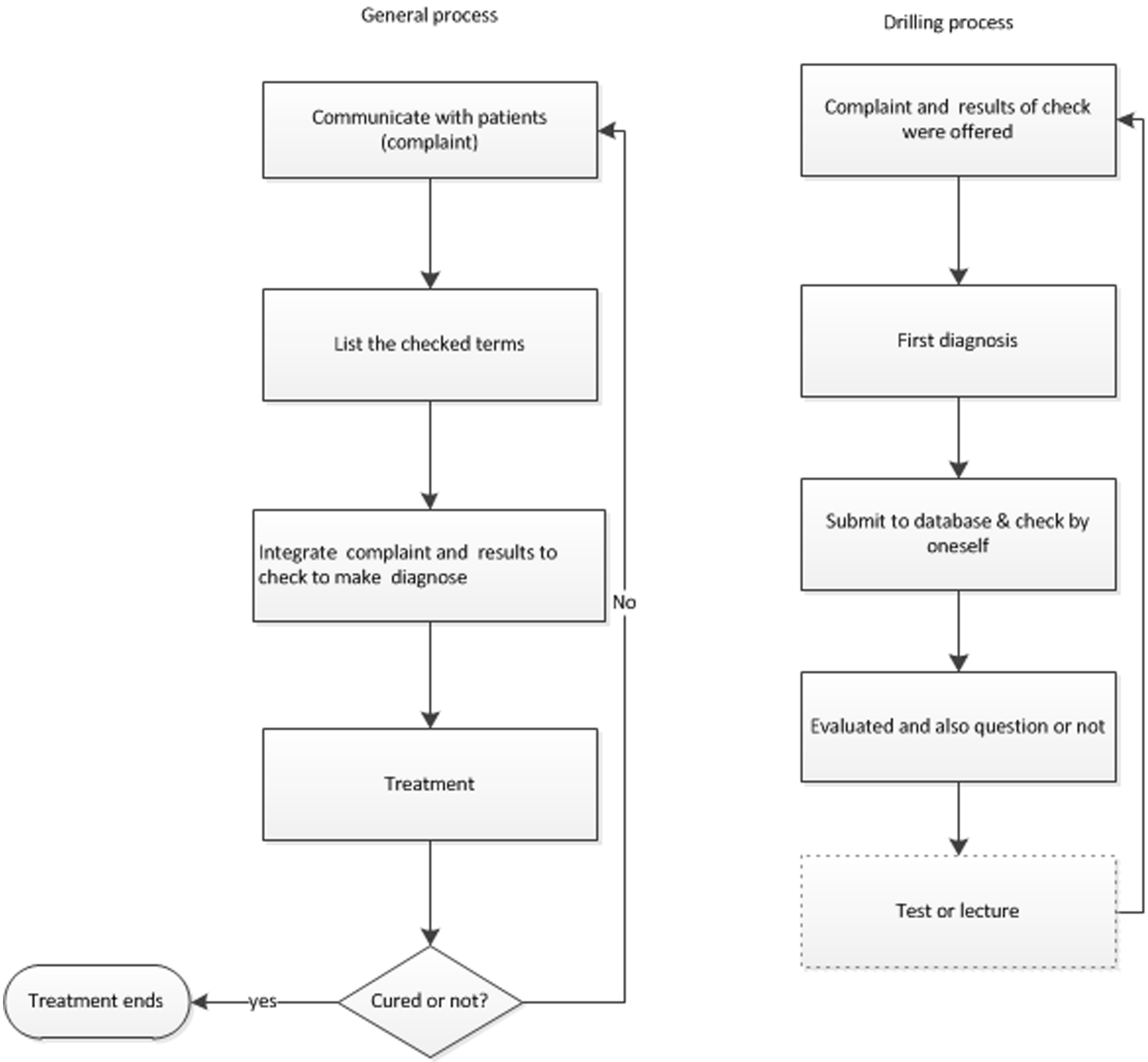

Figure 1. Drilling process and the general process: action in the dotted box occurred at some time or not, which is decided by tutors.
doi:10.1371/journal.pone.0093889.g001

group differences in post-test scores using the first-score as a covariate. Finally, a generalized mixed-effort model using a group, TP and a group × TP interaction as a fixed effect, and intercept as a random effect were used to compare the trajectory change in test scores over time between groups. Statistical analyses were performed using SPSS version 17.0 (SPSS Inc., Chicago, IL, USA). Statistical significance was set at a p value ≤ 0.05.

Results

The participants who had missed one or more tests or who spent little time on the drilling training were expelled from the project. The final sample numbers was 15 participants in the training group and 21 in the control group. One participant was added to the control group because he did not participate in any drilling but tested himself at each TP. Test scores at each TP were summarized using descriptive statistics including means and standard deviations (SDs) or means, maxima, and minima when datasets were not in a normal distribution.

The inter-rater reliabilities for the writing style, the accuracy of the image description, the accuracy of the diagnosis, and the correct treatment were 0.78, 0.79, 0.74 and 0.74 respectively. An internal consistency analysis of the test yielded a Cranach's alpha of 0.75.

Table 1 showed the scores of the style of writing for the two groups at each TP. Scores at the first TP represented the initial level of all participants. Before the intervention started, the control group and the drilling group did not differ significantly in scores ($t[34] = 0.46$, $p = 0.65$, $d = 0.16$). At each subsequent TP, the drilling group significantly outperformed the control group on the style of writing, even though the control group did also show improvement ($t[34] = 3.33$, $p < 0.001$, $d = 2.28$). The difference became less obvious at the third TP ($t[34] = 2.57$, $p = 0.02$, $d = 0.91$) and fourth TP ($t[34] = 1.50$, $p = 0.17$, $d = 0.53$). At the fifth TP, the difference between the two groups was significant ($t[34] = 4.21$, $p < 0.001$, $d = 1.51$). Using the first-score as a covariate, the ANCOVA also showed that except 4th TP, the drilling group significantly outperformed the control group from the 2nd to 5th TPs ($F[1,33] = 66.5$, $p < 0.0001$; $F[1,33] = 8.10$, $p = 0.008$; $F[1,33] = 1.99$, $p = 0.17$; $F[1,33] = 18.10$, $p < 0.001$).

Table 1 also showed the scores on the accuracy of the image description for the two groups at the five times points. The groups did not differ significantly in score until the 3th TP ($t[34] = 0.77$, $p = 0.44$, $d = 0.27$; $t[34] = 0.87$, $p = 0.39$, $d = 0.31$; $t[34] = 0.27$, $p = 0.79$, $d = -0.09$). In the subsequent TPs, the drilling group outperformed the control group at the 4th TP ($t[34] = 2.08$, $p = 0.04$, $d = 0.73$) and 5th TP ($t[34] = 3.10$, $p = 0.004$, $d = 1.11$). Using the first-score as a covariate, the ANCOVA showed that the groups did not differ significantly in score until the 4th TP ($F[1,33] = 0.12$, $p < 0.73$; $F[1,33] = 0.52$, $p = 0.48$; $F[1,33] = 3.99$, $p = 0.054$). The drilling group outperformed the control group at the last TP($F[1,33] = 3.99$, $p = 0.005$).

Scores on the accuracy of the diagnosis were displayed in Table 1 for the two groups at the five TPs. The difference between the two groups was significantly obvious except the 1st TP. However, the control group outperformed the drilling group at the 2nd TP. Then he drilling group significantly outperformed the control group until the 5th TP. Similar results were found using the ANCOVA whose covariate was the first-score.

Scores on the correct treatment selection in Table 1 showed the differences between the two groups started to become significant at the 3rd TP. At the end of the drilling project, the scores of the drilling group were much higher than the control group, which were consistent with the results yielded by the ANCOVA which used the first-score as the covariates.

Results of the sum of scores differed through the course. The difference in the beginning was the result of the difference amassed from the 4 parts.

As Figure 2(a) showed, after the introduction of the intervention, the drilling group showed a significant increase in the style of writing ($d = 1.43$, $p < 0.001$). However, a slight decrease occurred afterwards ($d = -0.25$, $p = 0.23$). As the amount of practice increased, the progress of the drilling group was evident at subsequent TPs. The control group progressed steadily on the writing style throughout the study, except at the last TP. However, their overall progresses were not far from the initial state for the control group ($d = 1.49$, $p < 0.001$), compared to the drilling group ($d = 2.01$, $p < 0.001$). The group × time interaction effect in the mixed-effect model was significant.

Compared to the trends of the style of writing scores, the trends on the accuracy of image description of both groups shown in Figure 2(b) were similar; both groups progressed steadily at the first four TPs but regressed at the final TP. However, the overall progress of the drilling group was better ($d = 2.56$, $p < 0.01$) than the control group ($d = 1.22$, $p < 0.01$). The group × time interaction effect in the mixed-effect model was less significant than for the writing counterpart.

The performance scores of both groups for the accuracy of diagnosis displayed in Figure 2(c) were much different. The changes in the control group scores progressed steadily at the first four TPs and retrogressed at the final TP. The change in the drilling group scores was not significant at the second TP ($d = 0.11$, $p = 0.59$). After that, the improvement was obvious. The final scores of the drilling group were much better ($d = 12.51$, $p < 0.0001$) than the control group ($d = 2.21$, $p < 0.001$). The group × time interaction effect in the mixed-effect model was significant ($p < 0.0001$).

The trends for the correct treatment selection were similar to those for the accuracy of diagnosis (Figure 2(d)). The drilling group showed great performance at the third TP ($d = 1.47$, $p < 0.001$). Though the effect of size on the values at each TP in the control group was larger than the ing group, the overall progress was not larger. The final change ($d = 2.08$, $p < 0.001$) in the control group was less than that in the drilling group ($d = 11.76$, $p < 0.0001$). The group × time interaction effect in the mixed-effect model was significant.

Discussion

Besides China, many other developing countries also faced the similar inequality of physician distribution [21–23]. The inequality is not only reflected in the physicians number but also in the diagnostic skills of physicians. Therefore, the CME of rural physicians needs additional intervention. In this study, An on-the-job drilling schema was designed for the rural physicians, junior physicians, or physicians who did not have a strong educational background. Results showed that applying the design principle based on the training schema improved rural physicians' skills in diagnostic radiology, including the style of writing reports, the accuracy of event description, the accuracy of diagnosis, and the correct treatment selection. The detailed changes in each part were different. At the initial stage, the writing style in the training group showed a considerable amount of progress. It seemed that the writing style could be improved in a short time, because it required less basic knowledge. The accuracy of image description improved the least among the four areas. The accuracy of image description requires not only the strong knowledge in medicine but also more strict knowledge in radiology terminology. Image description needs more time to show a large amount of progress.

In the early period, participants in the training group hardly made the correct diagnosis. Gradually, they started to know checking back illness condition of DSPs. Therefore, the diagnoses became more accurate. However, some physicians became overconfident; and made some mistakes. The tutors recognized the issue and helped them to rectify those problems. In cases of equivocal medical radiographic images or where the features were not obvious, the physicians did not know how to use more advanced equipment to support the diagnosis at the beginning. After the physicians did know how to use advanced equipment, such as CT, they often suggested their use in a wrong situations. Under the tutors' guidance, the physicians gradually learned the right suggestion for advanced testing. Furthermore, by combining the image information with the physical status of the patient, the physicians selected more appropriate treatments. In the other side, the amount of change on the four components in the control group was similar. The internal differences within the control group were smaller than those in the training group. Because of the limitation

Table 1. Scores of the two groups at five TPs.

	The first TP		The second TP		The third TP		The fourth TP		The fifth TP	
Style of writing	drilling group(n = 15)	control group(n = 21)	drilling group(n = 15)	control group(n = 21)	drilling group(n = 15)	control group(n = 21)	drilling group(n = 15)	control group(n = 21)	drilling group(n = 15)	control group(n = 21)
Mean±SD or Mean(Min,Max)	0.52±0.09	0.51±0.10	0.80±0.12	0.60±0.09	0.75±0.12	0.67±0.09	0.79±0.15	0.73±0.10	0.83±0.15	0.61±0.09
t-test or rank sum test(P value)*	0.65		<0.001		0.02		0.17		<0.01	
ANCOVA test@	-		<0.0001		<0.0001		<0.0001		<0.0001	
Cohen's d^{Ψ}	0.16		2.28		0.91		0.53		1.51	
Accuracy of the image description										
Mean±SD or Mean(Min,Max)	0.46±0.09	0.46±0.10	0.53±0.07	0.54±0.08	0.57±0.12	0.58±0.09	0.73±0.10	0.68±0.07	0.71±0.13	0.59±0.08
t-test or rank sum test(P value)*	0.44		0.39		0.79		0.04		0.004	
ANCOVA test@	-		0.73		0.48		0.41		0.02	
Cohen's d^{Ψ}	0.27		0.31		−0.09		0.73		1.11	
Accuracy of the diagnosis										
Mean±SD or Mean(Min,Max)	0.15±0.04	0.15±0.04	0.16 (0.11,0.20)*	0.22±0.03	0.38±0.08	0.3±0.04	0.57±0.09	0.38±0.04	0.75±0.12	0.35±0.06
t-test or rank sum test(P value)*	0.90		<0.001		<0.001		<0.001		<0.001	
ANCOVA test@	-		<0.0001		<0.001		<0.0001		<0.0001	
Cohen's d^{Ψ}	0.04		−2.34		1.78		3.62		4.96	
Correct etreatment selection										
Mean±SD or Mean(Min,Max)	0.30±0.03	0.29±0.05	0.38±0.07	0.38±0.03	0.62±0.10	0.45±0.03	0.71±0.15	0.53±0.05	0.85±0.17	0.49±0.04
t-test or rank sum test(P value)*	0.50		0.61		<0.001		<0.001		<0.001	
ANCOVA test@	-		0.86		<0.0001		<0.0001		<0.0001	
Cohen's d^{Ψ}	−0.23		−0.2		2.27		1.72		3.44	
Sum of scores										
Mean±SD or Mean(Min,Max)	1.59±0.17	1.40±0.15	1.88(1.55,2.24)*	1.72±0.13	2.32±0.10	1.6±0.15	2.80±0.37	1.9±0.17	3.18±0.39	1.82±0.14
t-test or rank sum test(P value)*	<0.01		<0.01		<0.001		<0.001		<0.001	
ANCOVA test@	-		0.22		<0.001		<0.0001		<0.0001	
Cohen's d^{Ψ}	1.28		1.14		6.45		2.06		3.68	

*Two sample t-test or two sample rank sum test.
@ANCOVA test adjusted by the first-score as a covariate.
$^{\Psi}$Cohen's effect size.
doi:10.1371/journal.pone.0093889.t001

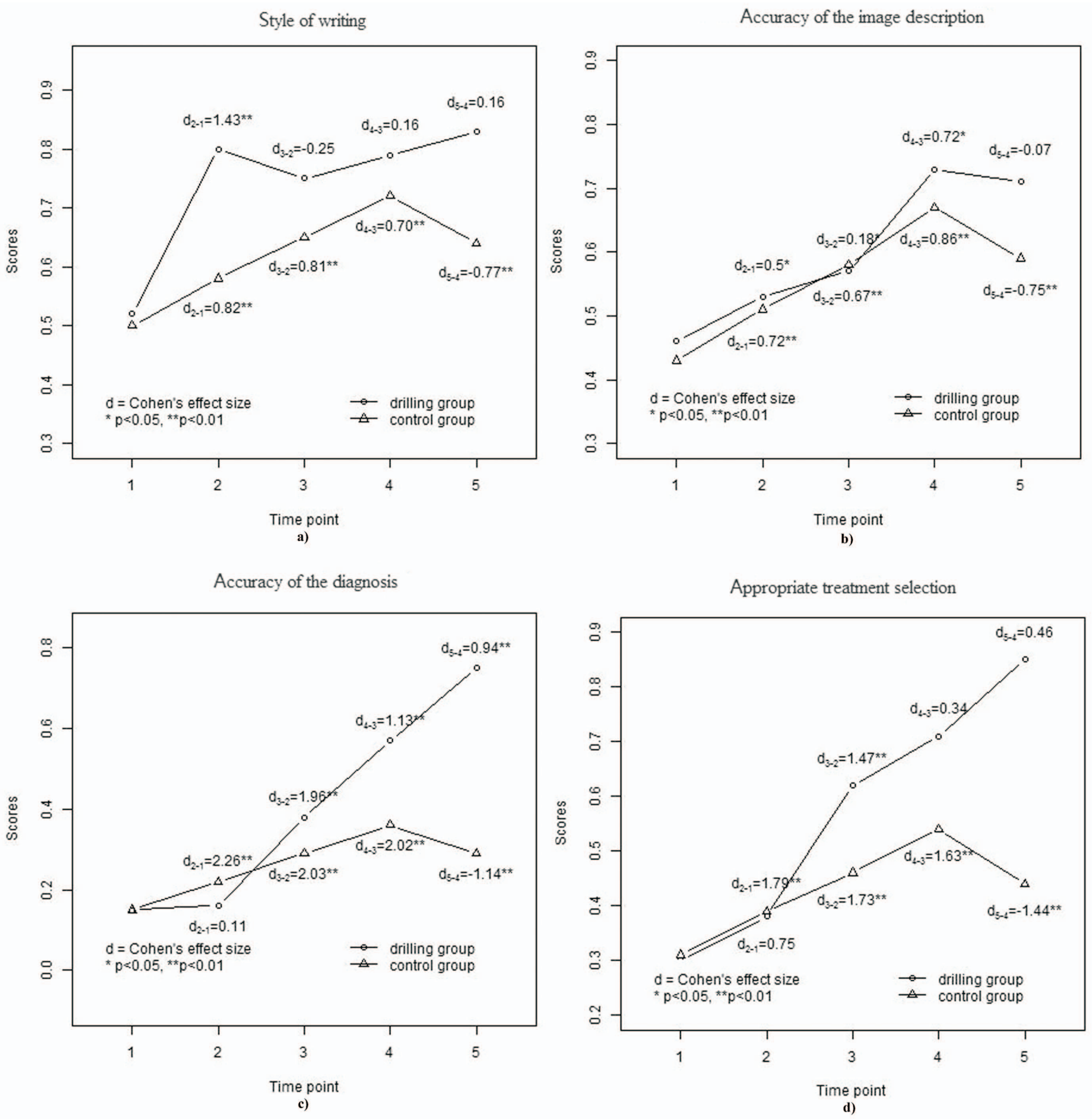

Figure 2. Results and change on a) the style of writing, b) the accuracy of the image description, c) the accuracy of the diagnosis and d) the appropriate treatment selection plotted by group over time.
doi:10.1371/journal.pone.0093889.g002

of local resource, physicians in the control group learned little knowledge about radiologic images. Once these images appeared in the test, most participants in the control group made mistakes. Some serious diseases might be diagnosed as less serious conditions and an incorrect therapy would be recommended. This would delay appropriate treatment, even cause medical accident.

There were several limitations to the current study. These results just illuminated the time point when participants gain more progress than counterparts did. The time span for a skilled to be learned much by one individual cannot be estimated exactly. In our trial, it is very hard to tell the minimal time span for a usual case to be learned or mastered. The study sample was small because it was difficult to persuade more rural physicians to participate in this trial for nearly one year. This study only focused skills in diagnostic radiology that helped to diagnose some pulmonary diseases, but these indices were easy to be confused. The effectiveness of these lessons would be assessed in other medical departments to further support our conclusion. Though the time spent on the drilling by the participants in the training group was recorded with help of information technique, the real

situation was hard to catch. Hence, the analysis between performance and time-used was incomplete. In the tests, we also found that there were some communications among participants of two groups. It seemed that these communications might increase the scores of the control group, but an all-round progress still required a systematic and consistent training process.

In the beginning of the study, we were concerned about the lack of motivation in the rural physicians, but it was not seen in the early stages. As the physicians made some advancement, their attitudes became more positive. The positive attitudes were seen among young physicians in both groups. The assistance from the tutors was a positive factor and could not be ignored. Another motivation was the long-term support from local governments.

Besides the introduction of new concepts and techniques in medical education in the rural hospitals, a reasonable policy must be constructed in order to support the appropriate intervention. The similar viewpoints were also expressed by some rural physicians and managers of rural hospital. Highly efficiency intervention must be supported by an optimization of techniques and policies.

Conclusion

An on-the-job training schema was designed to help rural physicians improve their skills in diagnostic radiology. It could be accepted by these physicians in a short time, and the skills in diagnostic radiology of rural physicians can be improved effectively. After one year's drilling, participants can write diagnosis report and describing images rightly, making a relative right diagnosis and suggesting an acceptable treatment. The rural physicians would make quick and accurate diagnoses and send patients to the right place for care. Education plans designed for rural physicians must consider the educational background and work condition of these physicians. Besides increasing the amount of practical training, a regular tutor should be also appointed to help these physicians via internet or in other ways as well. The policy must be designed to match with the mode involved. An appropriate policy can improve the motivations of the learners, tutors and managers.

Acknowledgments

We appreciate the help from the students in radiology department of Shanghai Pulmonary Hospital affiliated to Tongji University and the managers in rural hospitals.

Author Contributions

Conceived and designed the experiments: CJ. Performed the experiments: XS TF. Analyzed the data: TF. Contributed reagents/materials/analysis tools: XS WN TF HW. Wrote the paper: XS CJ TF HW.

References

1. Dollar D (2007) Poverty, inequality, and social disparities during China's economic reform. World Bank Policy Research Working Paper.
2. Jiangang P, Lee B-S, Guanzheng L, Jing H (2008) Regional Economic Disparity, Financial Disparity, and National Economic Growth: Evidence from China.
3. Meng Q, Zhang J, Yan F, Hoekstra EJ, Zhuo J (2012) One country, two worlds–The health disparity in China. Global Public Health 7: 124–136.
4. Ganatra HA, Bhurgri H, Channa R, Bawany FA, Zafar SN, et al. (2009) Educating and informing patients receiving psychopharmacological medications: are family physicians in Pakistan up to the task? PloS one 4: e4620.
5. Tienan F, Xiwen S, Chenghua J (2012) Discussion on Distance Drilling Pattern of Doctors in Rural Area Based on Digital Standardized Patient. Scientific Journal of Education Technology 2: 7–12.
6. Hamm MP, Klassen TP, Scott SD, Moher D, Hartling L (2013) Education in Health Research Methodology: Use of a Wiki for Knowledge Translation. PloS one 8: e64922.
7. Rowe AA, Bonham AJ, White RJ, Zimmer MP, Yadgar RJ, et al. (2011) CheapStat: An Open-Source,"Do-It-Yourself" Potentiostat for Analytical and Educational Applications. PloS one 6: e23783.
8. Li X, Liu J, Huang J, Qian Y, Che L (2013) An analysis of the current educational status and future training needs of China's rural doctors in 2011. Postgraduate medical journal 89: 202–208.
9. Apolinario D, Brucki SMD, de Lucena Ferretti RE, Farfel JM, Magaldi RM, et al. (2013) Estimating Premorbid Cognitive Abilities in Low-Educated Populations. PloS one 8: e60084.
10. Smith ZJ, Chu K, Espenson AR, Rahimzadeh M, Gryshuk A, et al. (2011) Cell-phone-based platform for biomedical device development and education applications. PLoS One 6: e17150.
11. Harden RM (2005) A new vision for distance learning and continuing medical education. Journal of Continuing Education in the Health Professions 25: 43–51.
12. Issa N, Mayer RE, Schuller M, Wang E, Shapiro MB, et al. (2013) Teaching for understanding in medical classrooms using multimedia design principles. Medical education 47: 388–396.
13. Ruiz JG, Mintzer MJ, Leipzig RM (2006) The impact of e-learning in medical education. Academic medicine 81: 207–212.
14. Frehywot S, Vovides Y, Talib Z, Mikhail N, Ross H, et al. (2013) E-learning in medical education in resource constrained low-and middle-income countries. Hum Resour Health 11: 4–11.
15. Chib A, Phuong TK, Si CW, Hway NS (2013) Enabling informal digital guanxi for rural doctors in Shaanxi, China. Chinese Journal of Communication 6: 62–80.
16. Peled JU, Sagher O, Morrow JB, Dobbie AE (2009) Do electronic health records help or hinder medical education? PLoS medicine 6: e1000069.
17. Curran VR, Fleet L (2005) A review of evaluation outcomes of web-based continuing medical education. Medical education 39: 561–567.
18. Buck G (1990) Development of simulators in medical education. Gesnerus 48: 7–28.
19. McGaghie WC, Issenberg SB, Petrusa ER, Scalese RJ (2010) A critical review of simulation-based medical education research: 2003–2009. Medical education 44: 50–63.
20. Krousel-Wood M, He J, Booth M, Chen C-S, Rice J, et al. (2012) Formal public health education and career outcomes of medical school graduates. PloS one 7: e39020.
21. Khan AR (2012) Public Health Posting As a Motivating Factor for Medical Students to Work in Rural Areas upon Graduation. Journal of Education and Practice 3: 233–238.
22. Doherty J, Couper I, Fonn S (2012) ISSUES IN MEDICINE: Will clinical associates be effective for South Africa? South African Medical Journal 102: 833–835.
23. Vyas R, Zachariah A, Swamidasan I, Doris P, Harris I (2012) A Networking Approach to Reduce Academic and Social Isolation for Junior Doctors Working in Rural Hospitals in India. Education for Health 25: 70.
